# Neuralgia

**Published:** 1872-11

**Authors:** H. M. Grant


### SELECTED ARTICLES.

### ARTICLE IV.

### *Neuralgia.*

An Essay read before the Abingdon Academy of Medicine on the 4th of March, 1872.

BY H. M. GRANT, M. D., D. D. S.

*Mr. President and Gentlemen*
*of the Abingdon Academy of Medicine:*

For the first time, I, this evening, have the honor of appearing before you in the capacity of an essayist, and with an eye ever directed to the investigation and discussion of such practical subjects as are best suited to our Society gatherings, and to comply with the request of our honored President, seconded by a resolution adopted by this Academy at our last meeting, I have chosen as my subject, for a short essay, NEURALGIA—which literally means *pains in nerves*—a painful affection of the nerves. The particular designation of neuralgia is determined by the situation of the affection, as Neuralgia Faciæ, or Tic Douloureux, when it affects the branches of the fifth pair of nerves. Neuralgic affections, with, perhaps, the exception of simple fevers, are among the most frequent and most tormenting of human ailments, and no diseases can be named that are more perplexing to the physician, and respecting whose essential nature they have more discordant theories, or more various, and even opposite methods of treatment.

Neuralgic or nervous pain, is not only a symptom of almost all acute and most chronic diseases, but also a distinct affection of the nerves themselves. To this latter the term neuralgia in its strictest sense is or should be applied. It may have its seat in any of the nerves of common sensation, and in some instances affect those of *organic*

life. Neuralgia may arise from many causes; sometimes no cause can be discovered, during life or after death; in which case the disease is attributed to a change in the condition of the nerve itself.

In other instances it is the consequence of a debilitated state of the system, and follows prolonged lactation, long continued and excessive discharges, or exhaustion from loss of blood. It also occurs in anæmia, etc., etc.

I need not occupy your time with the numerous details of the various classifications. The neuralgia to which I call your attention in this paper is, as I shall denominate it, *Dento-neuralgia*, or those nervous pains experienced in remote parts of the body; in the ear, temples, scalp, the muscles of the neck, and intercostal muscles; in the shoulders, arms, fingers, chest, heart, liver, womb, back, loins, calves of the legs and toes; superinduced by an irritable condition of the dental system.

Of the many divisions into which the general pathology, etiology, &c., of the organs of the animal system have been divided, none have met with so little attention or investigation as the special pathology of the nerves of the dental apparatus. The great misfortune of the medical profession has been, that these dento-neuralgic affections in their connection with dental medicine do not pay the physician as well as his manipulations in a case of surgery, or obstretrics. Hence, with a very few exceptions, the general practitioner of medicine finds no charms in the study of medico-dental philosophy, so essential to the accomplished physician. He chiefly expends his erudition in solving the problem, which is the most professional and scientific, the attending of a case of syphilis, or setting a broken bone. As the medical world, as a whole, comprehensively know but little of dento-neuralgic disease, it, of course, receives the go-by, and is left to be treated by the family dentist, (if such there be.) The treatment indicated has, therefore, been inadequate to the case, as the diagnosis, implicating parts as being idiopathically, which have been really symptomatically and sympathetically affected.

The ever-recurring attempt of offering a pseudo principle, with here and there a glimmer of an idea of a remote and exciting cause, without a fact to demonstrate or explain the theory, has been all that has ever been afforded to medical and dental science to enlighten and elucidate the real nature of the lesion exciting the various complex characteristic of dento-neuralgic derangements implicating sympathetically various and distinct regions of the body.

It is true that many distinguished medical men have arduously employed their knowledge in the investigation of neuralgic phenomena; but to this period, so far from affording any insight for our government in their treatment, or throwing any light upon this important and interesting subject, their efforts to elucidate the causes of neuralgic diseases have tended rather to render them still more obscure.

I have consulted in vain medical, surgical, and medico-dental literature upon the subject of dento-neuralgic affections. Prof. Harris (whose name I almost revere) in his "Dictionary of Dental Science," after all his research only furnishes the definitions of writers upon odontalgia.

Mr. Thos. Bell, of English notoriety, says: "It not unfrequently happens that parts remote become the apparent seat of pain from the exposure of the nerve of a tooth."

Here you will observe that Mr. Bell only refers to exposed nerves. He does not appear to think or suppose that dento-neuralgic sympathies are produced otherwise.

The eminent author, Dr. Good, says "neuralgia often is an idiopathic affection, dependent upon a peculiar irritation from a cause we cannot trace. It is more frequently a disease of sympathy produced by pregnancy, rheumatism or acrimony of the stomach."

The distinguished Dr. Wood speaks of neuralgic affections from the teeth, only when the affection is marked by pain in the teeth.

Prof. Bond, in his treatise on Dental Medicine, says: "It is not certain whether the seat of the disease is in the neurilemma or in the nervous pulp.

"It will at once be perceived that the physician must be called upon to discriminate between this disease and ordinary *toothache;* and unless he be properly informed upon these subjects, he may add to the terrible sufferings of his too confiding patient.

"In most cases the neuralgia of the jaw is at first taken for ordinary toothache, and ignorant, unscrupulous physicians, and dentists too, too frequently have extracted tooth after tooth; and have at last relinquished the patient to his or her aggravated sufferings." Prof. Bond then says: "From toothache depending upon exposed nerves, neuralgia may be diagnosed by this (self) evident centralization of the pain in a certain tooth; by the aggravation of it when the tooth in fault is touched, and by the positive evidence of a cavity in a tooth with an exquisitely sensitive pulp exposed."

Where such a simple odontalgic condition exists, it certainly needs no ghost to interest us. Now, I have often met with teeth, with the pulp exposed and exceedingly tender to the touch, exposed to all external agents, yet no pain whatever was experienced in these teeth; but sympathetic pains existed elsewhere. This especially refers to the "dentes supientiæ" or wisdom teeth, in which, in nine cases out of ten, if the nerve is exposed and exquisitely tender to the touch, the pain is experienced in the teeth, jaws, and face, anterior to the wisdom teeth, with neuralgic affections of the ear and temples, with nervous headache, muscular neuralgic pains in the scalp, neck, and various parts of the body.

And I would here remark that often, very often, it is diseased wisdom teeth which causes "the extraction of tooth after tooth," and still the torturing pain experienced by the patient continues without any relief being obtained, from the simple fact that the pain is almost invariably experienced in the teeth anterior to the wisdom.

We need not wonder or be surprised at this condition of things when we remember "that the nerves that are distributed to the teeth, and ear, have a common origin in the branches of the fifth and seventh pairs of nerves, while the immediate nervous relation of the auditory apparatus with the cerebral mass, and its great covering, the dura mater, is strikingly apparent."

The following illustrative cases of dento-neuralgic sympathies are on record. The great Dr. Rush mentions a case of "madness occasioned by diseased teeth which were no ways painful to the patient, and therefore offered no diagnostic mark that the teeth were the proximate and exciting cause of the mania affecting the patient."

Dr. Rush also records "a supposed case of hip joint disease, complicated with rheumatic affections, being immediately removed after the extracting of a tooth, which proved to be the exciting cause of the sympathetic affections of the hip-joint and adjacent parts."

The *London Lancet* records a case of neuralgia of the womb, immediately disappearing after the extracting of a diseased tooth.

The distinguished dentist of Philadelphia, Dr. Kœcker, relates a case of epilepsy, at once disappearing after the extraction of some diseased teeth.

I will also relate the circumstances attending a case which came under my own observation, in 1855, in the city of Knoxville. A young gentlemen of about 23 years of age called at my office to consult me about the extraction of some of his back teeth, which I found very badly decayed, some of them broken off down to the gums and alveolar process. He remarked to me, upon taking my chair, that his cheek was swollen and stiff so much so that he could not smile, and that he had been advised by his medical advisor that his teeth were the cause. He was of a nervous, lymphatic temperament, with rather a fullness of the face, but of a white or pale appearance.

There was no pain attending the case; upon examination I found that he had partial paralysis of the muscles of the face, and advised him to have these diseased teeth extracted, which he did, and the paralysis was relieved in a very short time.

Still another case which came under my observation, in '68. A lady called at my office to consult me about her daughter's teeth, remarking at the time that she was very singularly affected, that she had a lower wisdom tooth on the right side that was very badly decayed, that it never ached, but that for several months past, when her monthlies made their appearance, that this tooth would get very sore and seemed to be longer than the other teeth, and that for two months past her regular catamenial had failed to come on. After making this statement of the case, she asked me if I supposed this tooth could have anything to do with the irregularity. I told her while this tooth might not be the exciting or proximate cause of her irregularly, that it would do her no harm to have it extracted. I extracted the tooth, and at her next period, as her mother afterwards informed me, she was relieved.

Hence, I must say, from my own observation, and from all I have been able to collate on this subject, that many, very many of the neuralgic derangements of different regions of the body can be traced, as their proximate cause, to the disordered condition of the dental system.

It is with this view, therefore, that I am induced to offer to this Academy this paper, to add to the not very extensive stock of information upon the subject of dento-neuralgic phenomena for the consideration of its members, with the hope that a full and free discussion of the subject, by this Academy, will be profitable to medical science.

For, as I said, in the beginning of this paper, few afflictions affecting the human family present themselves to the medical practitioner that occasions so many vexations, or produce so many perplexities and mortifications to professional feelings and pride, as does the unsuccessful treatment of these painful nervous derangements of the various parts of the body—known and accepted by the not very precise term of "neuralgic affections."

Nosologists have named neuralgic affections according to the locality of the nerves supposed to be the seat of pain.

Thus neuralgia facialis or tic douloureux, the tic convulsif of the French authors; neuralgic, policis sciatic; the ischias nervosum, or pain in the branches of the great sciatic nerves, &c., with other nerves.

Nineteen years of close observation and investigation has enabled me to conclude in my own mind as to the proximate cause of many neuralgic affections, demonstrating to my mind that a large number are sympathetic and symptomatic of dental derangements; but which nosologists have named in accordance with the region of the body in which the pain is experienced.

I have named these sympathetic neuralgic affections, dento-neuralgia, predicated upon the proximate cause, the peculiar condition of super or sub-excitement—irritation, if you please, of the dental nerves—the fifth pair of nerves—within and upon the bony tissues of the teeth and their maxillary bones and investing periosteum in their anastomosing connection with other sympathetic nerves; a morbid condition of the bony structure of the teeth, whether from original constitutional deficiency, or produced by vitiating gastric acids, or the premature decay of the teeth, dependent upon the absence of proper nutrition, or from scrofulous, scorbutic, or syphilitic diathesis, the action of mercury, arsenic, antimony, iron, quinine, iodides, &c., all of which exert their several peculiar influences upon the dental organs, and hence upon their nerves, causing dento-neuralgic symptoms in various parts of the body.

It is not my intention to detail the affections of the dental system in this paper; but will mention one or two exciting causes. We find the dental tissues irritated and changed in character from the causes I have already enumerated. And any one of you gentlemen may observe in many of the teeth which you occasionally may extract, the animal portion absorbed from the earthly constituents of the upper portion or orifices of the fangs, leaving them in a semi-transparent condition; the lower portions and necks to the crowns of the teeth running opaque and presenting a normal appearance. While this change is being produced in the fangs, the alveoli and the dental periosteum becomes exquisitely sensitive—moderately to cold or warm applications, but intensely acute to the presence of acids, sweetmeats and confectioneries. These are the primary effects of dental derangements and the premonitory symptoms of dento-neuralgic affections.

These teeth are often glassy, brittle, breaking readily under the extracting instruments. In many of the transparent fangs of such affected teeth, you will find the extreme points absent, from absorption ; impressing the uninitiated with an unfounded idea or belief that he has broken off the apex of the fang in the act of extracting the tooth; the sharp speculated points prove the contrary.

In the class of teeth whose crowns are worn down and their pulp chambers filled in by a deposit of osseous matter, amber-like dentine, we too often find the maxillary absorbents exercising their baneful influence upon the roots of the altered teeth, with recession of the gums and their alveoli. Medical practitioners, and alas! some dental practitioners too, designate these affections "scurvy in the gums."

The absence too of antagonizing teeth is another exciting cause of dento-neuralgic affections. The lower jaw, or more properly speaking "the inferior maxillary," finding no resistance, nothing upon which to rest, is by the contraction of the temporal muscles closed to an unnatural extent toward the superior maxillary, which, acting like an overshut door lacking the resistance of the proper door-post, overstrains its hinges; so the inferior maxillary bone overstrains its joints, affecting the nerve to an extent to superinduce nervous irritability and hence sympathetic neuralgic paroxysms.

The dental system thus affected, presenting no external physical disintegration (or decay,) exhibits some of the causes of dento-neuralgic affections in contra-distinction to odontalgic pains portrayed by Dr. Bond. Dento-nervous irritation being established in the dental system, which from

"the infant muling and puking in the nurse's arms" to the "last scene of all when death closes our earthly career," daily presents to our observation neuralgic, symptomatic, and sympathetic paroxysms.

Thus are daily presented to the medical world, the physician, the surgeon, and the dentist, innumerable instances in which each and all may recognize the essential starting point of a correct diagnosis of many rheums, pains, and convulsions, which, without any apparent exciting cause, experience shows to affect patients from childhood to old age, dependent upon and arising from a source often overlooked and even little dreamed of in medical and dental philosophy.

It is useless to cite the countless number of cases where patients have been subjected for years to the most harassing, and I might also say empirical, treatment in which have been used narcotics, sedatives, emollients, irritants, stimulants, counter-irritants, moxas, nervines, antispasmodics, sudorifics, phosphates, chalybeates, alkalies, acids, arsenic, mercury, copper, bismuth, iodine, potassa, quinine.

Then again have been brought to bear phlebotomy, cupping and leeching, alteratives and aperients, cold and hot baths, sulphur baths and fumigations, electricity, sea-bathing, the surgeon's knife, for the excision of some innocent, unoffending ganglionic centre.

Then again have been called into requisition homœpathy, hydropathy, spiritualism, polorization, clairvoyance, thomp sonianism, and nostrums, have all stepped in and taken their turn, " certain to effect a cure." If the now wretched, patient sufferer has had sufficient stamina, or vital force, to resist these terrible onslaughts and assaults upon the citadel so frail—so wonderfully and fearfully made—it has been to sink into helplessness and despair.

Yet, gentlemen of the Abingdon Academy of Medicine, I say that many, very many of these severe cases might, with the proper diagnosis first, and then the simple application of the forceps or other proper instrument in the hands of a skillful manipulator, be relieved in a way and manner not dreamed of by too many of us. I said with the proper diagnosis first. Let us see what this proposition means. I quote from Dr. Richardson, of London.

The point of diagnosis then is simply this: When called to a patient suffering with neuralgia, if no other external case presents itself, we must then look to the dental apparatus for the proximate cause. This arises, not from anything specific in dental disease, but from the circumstances that a tooth, as connected with the periphery of the nerve, is more subjected than other parts to such diseases of its own structure as shall expose the nerve periphery to exertion.

"In a case where toothache in its most severe form occurs, from either exposure of the supplying nerve or injury, or pressure, in such case the toothache is a true neuralgia—localized, certainly, but still a neuralgia.

"But neuralgia having a local origin and a more extended range, may have a tooth for its centre in a way different to that which has been described."

In such cases the tooth need not itself, in its crown part, be diseased, nor need its nerve be exposed. The disease lies deeper; still lies often in the extreme point of the fang, and thus, by injuring the nerve at its very outset from its parent trunk, and by injuring sometimes the trunk itself, the tooth distributes by reflection, or I had better say by conduction of excitation, the pain to the nerve supplies of all surrounding structures.

"The pure 'tic' from a diseased tooth is thus produced, and in other parts of the body a neuralgic circle may have a similar causating centre, but this one illustration, as being the most familiar will suffice.

"The points of diagnosis in cases of this character are exceedingly important, and in such points of diagnosis the dentist is often more expert than either physician or surgeon. Without any pretense of being more conversant than my compeers with this interesting diagnostic history, I cannot let the opportunity pass of stating, in a few words, by what common sense principles we should be guided to insure success in deciding on diagnosis in this form of the neuralgic malady.

"If, then, a tooth be found carious, and the pain be referred by the patient to the tooth as the originating cause, and if this tooth is tender, and the touching of it intensifies pain, the common sense view is that the tooth is the cause.

"But if it happens, and this for reasons I have given is most commonly what does happen, an extended neuralgia is not traceable to a *carious* tooth; if the appearance of every tooth be sound, other and more refined diagnostic principles are required.

"In these instances the points to be ascertained are:

"1. Whether the neuralgia is confined to one side.

"2. Whether the patient is himself conscious or suspicious of a central cause in the teeth.

"3. Whether percussion of any tooth excites the pain.

"4. Whether the attack is brought on by exposure of the tooth to variations of heat or cold.

"5. Whether the pain is, or is not, intermittent.

"6. Whether remedies applied to the teeth are transitorily effective.

"Reasoning on these questions—if it be found that the neuralgia is confined to one side; if there is no distinct intermittence; if pressure on the nerve at its trunk reliever the pain; if local sedatives relieves the pain, and if the answer to the majority of the other queries be affirmative, the evidence is pretty clear that the cause is local, and that a diseased tooth is the local cause.

A very good test whether a neuralgia is or is not dependent on dental mischief deeply hidden, is to direct the patient, when the paroxysm is on, to take in his mouth and bring slowly in contact with the teeth, iced water. As the cold is first felt, the pain may or may not be increased, but after a time, as the benumbing effect of the cold tells on the tooth, the neuralgic pain, if the cause of it be local, will certainly be relieved for a period, and such relief is the clearest indication of the seat of the disease.

"In this form of neuralgia, if the diagnosis be skillfully made, there is more real success in treatment than in any other. The cause is removable."

And now, Mr. President and gentlemen, I have thus attempted to respond to the appointment of myself by this Academy to write a paper upon the subject of neuralgia as connected with the teeth, or rather dependent upon a diseased condition of the dental system. I assure you it is but a sense of duty which has compelled me to an exhibition of qualification and competency inadequate to the task.

If, however, I am incompetent to the task assigned me, I suppose it is justly chargeable to my own neglect, and want of application, and if in anything I have enunciated I have incurred your disapprobation, or that of the Fellows now present, I trust you will mingle justice with mercy, and deal generously with me. Once more allow me to thank you for the honor conferred upon me in my appointment as an essayist by the Academy and for your kind indulgence and attention.— *Virginia Clinical Record.*

---

